# Root Handling Affects Carboxylates Exudation and Phosphate Uptake of White Lupin Roots

**DOI:** 10.3389/fpls.2020.584568

**Published:** 2020-10-02

**Authors:** Raphael Tiziani, Tanja Mimmo, Fabio Valentinuzzi, Youry Pii, Silvia Celletti, Stefano Cesco

**Affiliations:** Faculty of Science and Technology, Free University of Bolzano, Bolzano, Italy

**Keywords:** carboxylates, cluster roots, phosphate uptake, root exudates, white lupin

## Abstract

The reliable quantification of root exudation and nutrient uptake is a very challenging task, especially when considering single root segments. Most methods used necessitate root handling *e.g.* root dissecting/cutting. However, there is a knowledge gap on how much these techniques affect root physiology. Thus, this study aimed at assessing the effect of different root handling techniques on the phosphate (P_i_) uptake and carboxylate exudation of white lupin roots. White lupin plants were grown hydroponically in a full and P_i_-deficient nutrient solution for 60 days. Phosphate uptake and carboxylate exudation of cluster and non-cluster roots were measured using custom made cells 1, 4, and 8 h after the onset of light. Three different experimental set-ups were used: i) without cutting the root apparatus from the shoots, nor dissecting the root into smaller root sections — named intact plant (IP); ii) separating the roots from the shoots, without dissecting the root into smaller sections — named intact root (IR); iii) separating the roots form the shoots and dissecting the roots in different sections—named dissected roots (DR). The sampling at 8 h led to the most significant alterations of the root P_i_ uptake induced by the sampling method. Generally, roots were mainly affected by the DR sampling method, indicating that results of studies in which roots are cut/dissected should be interpreted carefully. Additionally, the study revealed that the root tip showed a very high P_i_ uptake rate, suggesting that the tip could act as a P_i_ sensor. Citrate, malate and lactate could be detected in juvenile, mature and senescent cluster root exudation. We observed a significant effect of the handling method on carboxylate exudation only at sampling hours 1 and 8, although no clear and distinctive trend could be observed. Results here presented reveal that the root handling as well as the sampling time point can greatly influence root physiology and therefore should not be neglected when interpreting rhizosphere dynamics.

## Introduction

Phosphorus (P) shortage is one of the most limiting factors for agricultural production (van de Wiel et al., 2016) due to the low concentration and poor mobility of its plant-available fraction in soils. Furthermore, P fertilization efficiency is lower than 30% highlighting the urgent need to study the physiological and molecular adaptations of plants to the limited endogenous P availability of soils. In addition, due to the limiting natural resources available to produce P fertilizers and the ever increasing demand of food for a growing population, it is urgent to improve P utilization efficiency (PUE) to maintain agricultural production at its maximal extent (Veneklaas et al., 2012; López-Arredondo et al., 2014). With respect to the plant effects, P shortage can lead to a reduced photosynthesis (Rodríguez et al., 1998; Wissuwa et al., 2005; Turnbull et al., 2007), an increased root-to-shoot ratio (Fredeen et al., 1989; López-Arredondo et al., 2014), an enhanced growth of lateral roots and root hairs (Reymond et al., 2006; Péret et al., 2011), a reduction in phosphorylated metabolites (Müller et al., 2015) and an increased expression of proteins that do not require P as co-substrates (Cruz-Ramírez et al., 2006; Gaude et al., 2008). One of the most important adaptation strategies of plants to P shortage is the increased release of root exudates into the rhizosphere. Root exudates are commonly defined as root-derived primary and secondary metabolites characterized by both low (<1000 Da) and high (>1000 Da) molecular weight (Oburger and Jones, 2018; Pausch and Kuzyakov, 2018). These compounds are exuded either actively (through primary or secondary active transporters) or passively (through diffusion and/or exocytosis processes, or thanks to ionic channels - Jones et al., 2009; Vives-Peris et al., 2019). With respect to their chemical nature, these root exudates consist of a very wide range of compounds including carbohydrates (*e.g.* glucose, fructose), amino acids (*e.g*. alanine, glycine, glutamate), carboxylates (*e.g.* malate, citrate, oxalate, lactate) or secondary metabolites such as phenols, glucosinolates, vitamins, plant hormones, but also enzymes (*e.g*. phosphatases, phytases) and polysaccharides (*e.g*. pectic acid). The type and concentration of metabolites exuded by roots depend on many factors such as i) the plant genotype, ii) the environment (*e.g.* soil properties, temperature etc.) and iii) biotic or abiotic stresses (Jones et al., 2009; Baetz and Martinoia, 2014; Steinauer et al., 2016; Vives-Peris et al., 2019). The shortage of P mainly triggers the root release of phosphatases to mobilize the organic P pool, while carboxylates are mainly exuded in order to solubilize the nutrient from the inorganic fractions. In this regard, it is worth noting the role played by citrate and malate in root P acquisition by solubilizing and mobilizing *via* ligand exchange reactions the P bound to iron (Fe) or aluminum (hydro)oxides (Otani et al., 1996; Neumann and Römheld, 1999).

White lupin (*Lupinus albus* L., Fabaceae family) is a non-mycorrhizal plant, which is well known to exude huge amounts of carboxylates, especially under P starvation (Mimmo et al., 2011; Marschner, 2012; Valentinuzzi et al., 2015). Therefore, this species serves often as a model plant in studies of root induced mobilization of sparingly soluble soil P sources (Uhde-Stone, 2017). Under P deficiency some plant species of the Proteaceae family develop special root types, the so-called proteoid roots (or cluster roots), which are short lateral rootlets with a bottlebrush-like appearance (Dinkelaker et al., 1989). These cluster roots are extremely efficient in enhancing soil P availability due to the increase of the roots surface area by more than 100-fold and to the exudation of huge concentrations of protons, carboxylates and phosphatases, particularly in comparison with non-proteoid roots (Skene, 2000; Uhde-Stone, 2017). Furthermore, cluster roots also release large concentrations of flavonoids (*e.g.* genistein), probably to mobilize P complexed with Fe and to prevent microbial degradation of organic acids (Tomasi et al., 2008). It is interesting to note that the exudation of those compounds ensures the plant survival also in environments with basically no available P (Lambers et al., 2006). The *Lupinus´* cluster roots can be divided into distinct types based on their development stage: juvenile, immature, mature and senescent (Massonneau et al., 2001). The most active type of cluster roots in terms of carboxylate exudation is the mature one. Differently, the immature and juvenile rootlets are characterized for an abundant release of phenolic compounds (Weisskopf et al., 2006), whilst the senescent ones are mainly used for nutrient recycling (Massonneau et al., 2001; Wang et al., 2014). Moreover, there are some pieces of evidence showing that citrate exudation follows a diurnal and weekly pattern (Dessureault-Rompré et al., 2007; Tomasi et al., 2009; Mimmo et al., 2011). Even though the release of carboxylates has been widely described in literature, there is relatively limited information about the P uptake of the single cluster root types of white lupin. In this context, it is well known that P-deficient plants generally upregulate phosphate (P_i_) transporters in order to cope with the nutritional disorder (Raghothama, 1999). Moreover, it seems that the P uptake rate in the cluster roots is plant species dependent. In fact, Malajczuk and Bowen (1974) revealed that *Banskia grandis´* cluster roots have faster P uptake rates when compared to their non-cluster roots. The same was observed in *Protea compacta* and *Leucadendron uliginosum*, which both showed faster P uptake rates in their cluster roots (Vorster and Jooste, 1986). On the other side, *Banksia marginata* seems to have a slower P uptake rate in cluster roots than in ordinary roots (Lamont, 1982). Interestingly, in *Hakea prostata* the P uptake of cluster roots was similar to that measured in non-cluster roots having the same age (Shane et al., 2004). In this regard, it is important to note that a better understanding of root exudation and P uptake phenomena is crucial to improve the crop performance especially in P-limited conditions in order to maximize yield. However, the reliable quantification of exudation and nutrient uptake is a very challenging task, particularly when planned at the same time and in more realistic conditions. Root exudate sampling methods are usually d4ivided into i) soil-based and ii) hydroponically-based methods (Oburger and Jones, 2018; Vives-Peris et al., 2019). Soil-based methods may lead to more realistic results, albeit much more difficult due to the complexity to access the roots and sampling of the rhizosphere, as well as due to the degradation of exudates by soil microflora. Hydroponically-based experiments may be easier to perform, but plant development, physiology and root exudation are often affected by hydroponic growing systems since they are highly artificial environments (Valentinuzzi et al., 2015). Both methods usually necessitate root handling and, thus, an effect of this processing on the root physiology cannot be totally excluded (Oburger and Schmidt, 2016; Oburger and Jones, 2018; Vives-Peris et al., 2019). Indeed, little is known about how much the root handling can influence the root exudation and P uptake phenomena. Moreover, up to now most studies have been performed by using excised cluster roots without an analysis of the impact of this cutting on the phenomenon studied. For this reason, we developed a custom-made device to analyze different hydroponically grown cluster root tissue types without the need to excise the single cluster rootlets. In particular, this study aimed at assessing the effect of different root handling techniques on the P uptake and carboxylate exudation of white lupin roots at three different time points during the day (*i.e.* 1, 4, and 8 h after the onset of light). Three different experimental set-ups have been used: i) without separating the roots from the shoots, ii) separating the roots from the shoots, iii) separating the roots from the shoots and dissecting the roots in the different sections.

## Materials and Methods

### Plant Growth

Seeds of white lupin (*Lupinus albus* L. cv. Amiga; Südwestdeutsche Saatzucht, Rastatt, Germany) were germinated for 5 to 6 days in the dark at 22°C between two layers of filter paper, which were moistened with 0.05 mmol L^−1^ CaSO_4_. Homogeneous seedlings were selected and placed in black pots, containing either a full (control, C) or a P-free (-P) nutrient solution (NS). The NS had the following composition: 0.25 mmol L^−1^ KH_2_PO_4_ (not present in the –P solution), 5 mmol L^−1^ Ca(NO_3_)_2_ 4 H_2_O, 1.25 mmol L^−1^ MgSO_4_, 1.75 mmol L^−1^ K_2_SO_4_, 0.25 mmol L^−1^ KCl, 20 µmol L^−1^ Fe(III)-EDTA, 25 µmol L^−1^ H_3_BO_4_, 1.25 µmol L^−1^ MnSO_4_ 7 H_2_O, 1.5 µmol L^−1^ ZnSO_4_ 7 H_2_O, 0.5 mmol L^−1^ CuSO_4_ 5 H_2_O, 0.025 µmol L^−1^ (NH_4_)_6_Mo_7_O_24_ 4 H_2_O. The NS has been changed every 3 days. White lupin plants were grown in a climate chamber with the following conditions: day 14 h, 24°C, 70% relative humidity (RH); night 10 h, 19°C, 70% RH. The experiments were executed after 60 days of growth, when the cluster roots of the plants were fully developed.

### Visualization of Acidification

Qualitative visualizations of pH changes along the root axis were obtained using agar gel containing a pH indicator. Bromocresol purple (0.1 g L^−1^) adjusted to pH 6.2 with 0.1 mol L^−1^ NaOH was used as pH indicator. 1.8 mL of bromocresol purple was added in 500 mL liquid agar (0.75%, w/v). Selected roots were carefully placed in the pH indicator agar solution and incubated for 1 h at 25°C (Tomasi et al., 2013).

### Experimental Setup and Phosphate Uptake

**Figure 1:**
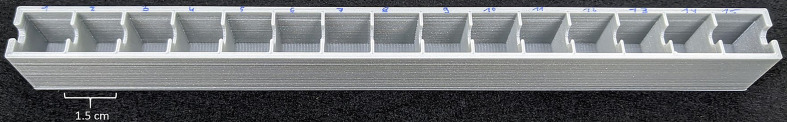
Custom made 3D printed device to measure phosphate uptake and root exudation of single cluster root tissue types with a temporal and spatial resolution.

**Figure 2:**
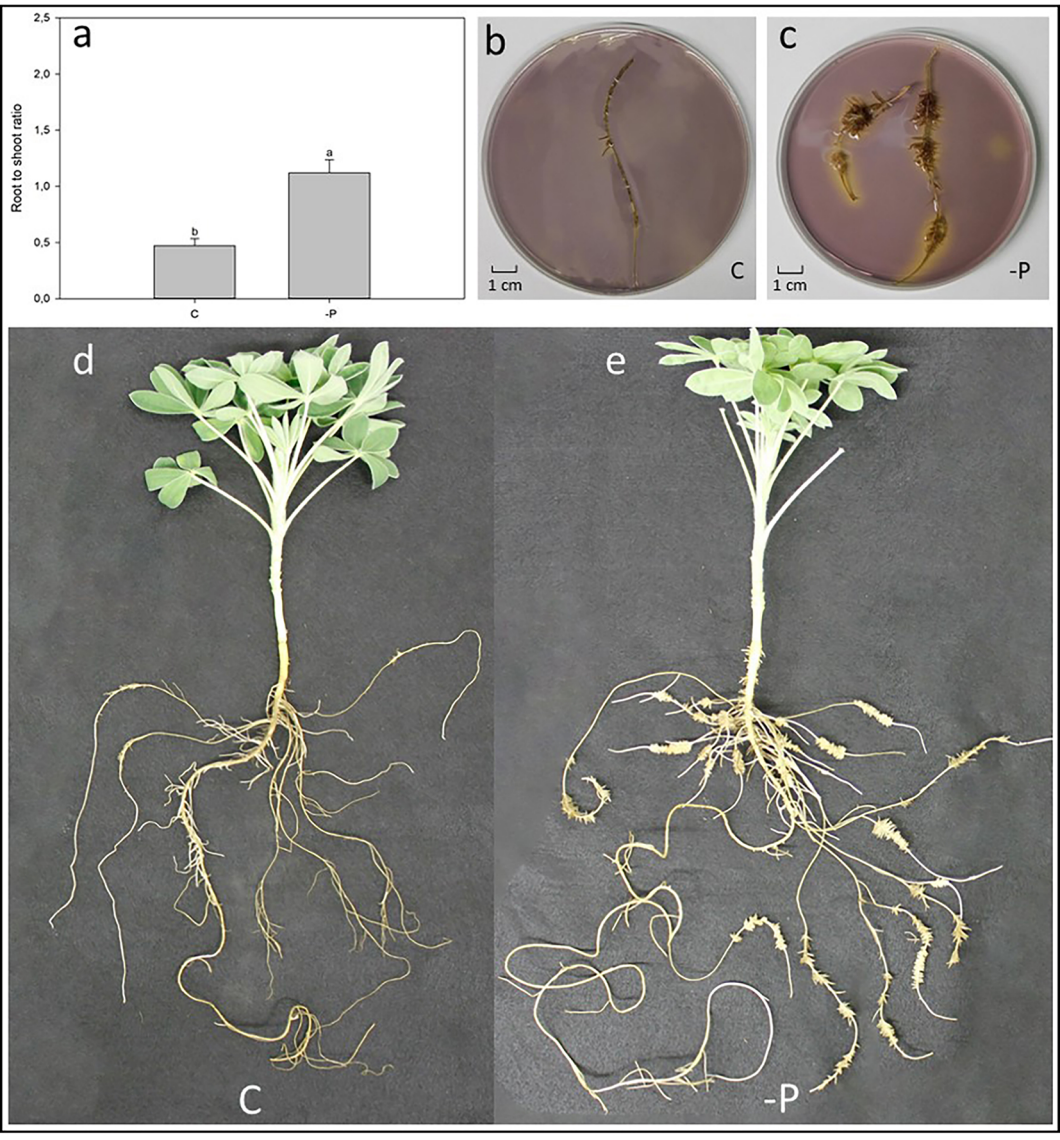
Effect of P deficiency on 60-day-old white lupin plants. **(A)** Root-to-shoot ratio of white lupin plants grown in either control **(C)** or P starvation (-P) conditions; data are presented as mean ± SE, n = 10; lower case letters above the vertical bars indicate statistical significance according to t-test with p < 0.001. **(B)** Acidification of a C white lupins’ cluster root laid in an agar gel mixed with pH indicator: yellow coloring indicates a lower pH than the purple color. **(C)** Acidification of a -P white lupins´ root laid in an agar gel mixed with pH indicator: yellow coloring indicates a lower pH than the purple color. **(D)** Representative white lupin plant grown in C condition. **(E)** Representative white lupin plant grown in -P condition.

We developed a custom-made device to study root exudation and nutrient uptake with a spatial and temporal resolution of hydroponically grown plants roots (**Figure 1**). The device was 3D printed out of polylactic acid (CREA 3D S.R.L., Italy) with a Ultimaker 3D printer. The device was 24.4 cm long, 1.9 cm wide and consisted of 15 (1.5 cm long x 1.5 cm wide) communicating cells (**Figure 1**). Roots of –P plants were divided in root apex (A), juvenile/immature cluster root (J), mature cluster root (M), senescent cluster roots (S), root parts without cluster root (R); whereas roots of C plants were divided into: roots parts without cluster (RC), cluster root like roots (C), control root apex (AC). We used three different experimental set-ups: i) without cutting the root apparatus from the shoots, nor dissecting the root into smaller root sections - named intact plant (IP); ii) separating the roots from the shoots, without dissecting the root into smaller sections– named intact root (IR); iii) separating the roots form the shoots and dissecting the roots in different sections—named dissected roots (DR) (**Figure 2**). In all the three different experimental set-ups, the roots were carefully placed inside the custom-made device in order to have the different root tissue segments as separated as possible. Previously, the roots were immerged in 0.05 mmol L^−1^ CaSO_4_ for 15 min and dried carefully with tissue paper. Every cell of the custom-made device was filled with 3.4 mL 100 µmol L^−1^ KH_2_PO_4_ uptake/root exudate collection solution. The roots were placed into the device and P_i_ uptake and root exudation were measured. The use of radiolabeled P_i_ was not possible since our laboratory is not allowed to use radioactive substances. The experiment was carried out three times during the day: 1, 4, and 8 h after the onset of light of the climate chamber. For all the three experimental set-ups and three sampling time points, we collected the root exudates after 1 h by pipetting 100 µl of uptake solution directly in a HPLC vial for further analysis. Fifty µl uptake solution were sampled after 15, 30, and 60 min and their P_i_ contents were analyzed. Phosphate uptake rate has been calculated by difference between the concentration of PO_4_^3-^ before and after the contact times of roots with the uptake solution. The values have been then normalized to the root fresh weight. The consumption of P_i_ in the uptake medium due to the root uptake was always less than 10%. After the collection of the solutions, the rootlets have been carefully dried with tissue paper and weighted. Phosphate concentration was determined colorimetrically according to the method of Murphy and Riley (1962). The method was scaled down in order to perform the reaction in a spectrophotometric cuvette with a final volume of 1 mL (50 µl of KH_2_PO_4_ standard, blank or sample, 5 µl *p*-nitrophenol,10 µl 2.5 mol L^−1^ sulfuric acid (H_2_SO_4_), 80 µl of the Murphey and Riley reagent and 855 µl deionized water). The adsorption was read at 720 nm after 10 min incubation at room temperature.

### Analysis of Carboxylates by High-Performance Liquid Chromatography (HPLC)

Carboxylates were separated on a cation exchange column (Aminex 87-H column, 300 × 7.8 mmol L^−1^, 9 μm, Bio-Rad) using an isocratic elution with 10 mmol L^−1^ H_2_SO_4_ as carrier solution at a flow rate of 0.6 mL min^−1^. Carboxylates were detected at 210 nm using a Waters 2998 photodiode array detector (Waters Spa, Italy). Standard carboxylates were prepared as individual stock solutions, using Sigma reagent grade compounds, and then combined to give diluted reference standards. Comparing the retention times of unknown compounds with those of pure compounds allowed the detection of carboxylates.

### Statistical Analysis

The results are presented as means ± standard error (SE). Statistical analysis was performed using SigmaPlot 12 on Windows 10 64 bit. Two conditions were compared through t-tests; p-values of < 0.05 were treated as statistically significant differences. Three and more conditions were analyzed by one-way analysis of variance (ANOVA) and means were compared using Holm-Sidak *post hoc* test at p < 0.05 to determine the significance of found differences.

## Results

### Root Morphology and Acidification

**Figure 3:**
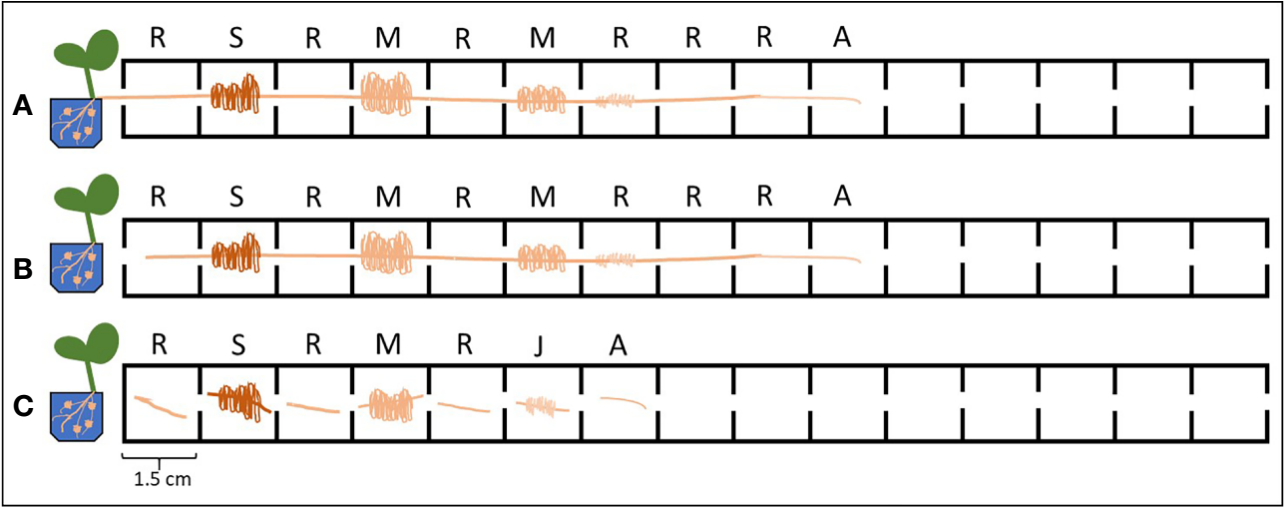
Different experimental set-ups used for the P_i_ uptake/exudation experiment. Letters above the cells indicate the root tissue type: R, root without cluster; S, senescent cluster root; M, mature cluster root; J, juvenile cluster root; A, apex. **(A)** Intact plants (IP): without cutting the root apparatus from the shoots nor dissecting the root into smaller root sections. **(B)** Intact roots (IR): separating the roots from the shoots, without dissecting the root into smaller root sections. **(C)** Dissected roots (DR): separating the roots from the shoots and dissecting the roots in different sections.

**Figure 3A** shows the root-to-shoot ratio of hydroponically grown white lupin plants, raised in control (C) and P deficiency (-P) conditions after 60 days. As expected, -P lupin plants showed a significantly higher (+42%) root-to-shoot ratio compared to C plants. Indeed, white lupin plants grown in -P developed huge amounts of cluster roots (**Figure 3E**), while plants grown control solution exhibited a much smaller root apparatus developing only very small amounts of cluster roots like root structures (**Figure 3D**). Furthermore, cluster roots of P-deficient plants acidified the rhizosphere as shown by the yellow color around the -P roots (**Figure 3C**). On the other hand, roots from white lupin plants grown in a full NS showed just a very limited acidification of the rhizosphere when compared to -P grown white lupin roots (**Figure 3B**).

### Phosphate Uptake

#### Phosphate Uptake After 1 h

**Table 1** shows the P_i_ uptake rate of C and -P roots 1 h after the onset of light in the climate chamber. The three different root handlings (IP, IR, DR) did not influence the P_i_ uptake in any of the two growing conditions (-P and C). On the other side, the P_i_ uptake was significantly affected by the different root segments within the same handling methods. In the intact plant (IP) handling method, the apex of -P roots (A) showed the highest uptake rate, while the lowest was found in mature cluster roots of -P plants (M) (A *vs* M +299%). In the handling method in which the roots were detached from the shoot but kept intact (IR), the significantly highest P_i_ uptake rate was found unexpectedly in apex rootlets of control plants (AC) and apex rootlets of -P lupins (A). On average the AC and A roots showed a 266% higher uptake when compared to all the other rootlets of both control and P-deficient plants. A similar trend could be observed when considering the completely dissected roots (DR) handling method. When the roots were completely dissected, the highest P_i_ uptake was found in AC, A, and RC rootlets.

**Table 1:** Phosphate uptake and citrate, malate and lactate exudation of white lupin roots grown in either -P or C conditions 1 h after the onset of light.

		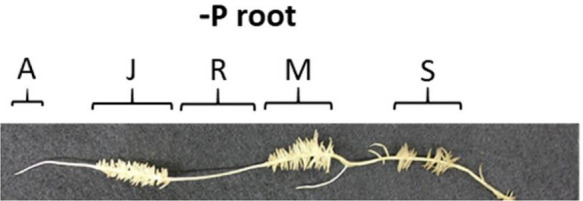 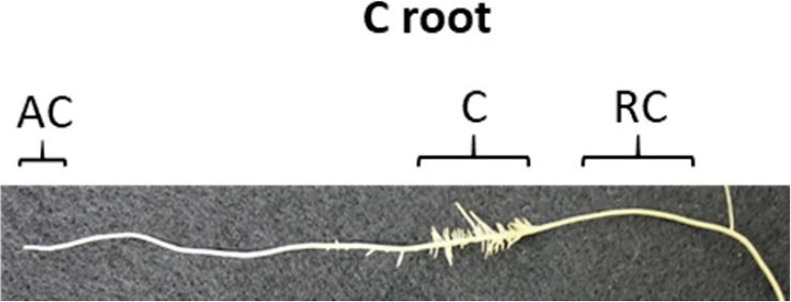								
1h	Sampling	Root segment								
		A	J	R	M	S	AC	C	RC	*p*
P_i_ uptake (µmol g^−1^ FW h^−1^)	IP	9.03 ± 3.69a A	4.18 ± 1.5abc A	5.15 ± 1.1abc A	2.26 ± 0.56c A	2.55 ± 0.77bc A	5.66 ± 5.3abc A	2.46 ± 0.85bc A	7.40 ± 1.24ab A	<0.001
	IR	14.33 ± 4.1ab A	0.92 ± 0.25cd A	8.73 ± 1.96bc A	2.50 ± 0.69cd A	0.79 ± 0.24d A	22.7 ± 1.33a A	7.84 ± 5bcd A	9.53 ± 1.86b A	<0.001
	DR	7.96 ± 0.97ab A	2.29 ± 0.72bc A	6.80 ± 0.43b A	1.21 ± 0.20c A	2.01 ± 0.61bc A	13.51 ± 4.12a A	4.64 ± 0.76bc A	10.78 ± 1.27a A	<0.001
*p*		ns	ns	ns	ns	ns	ns	ns	ns	
Citrate (nmol g^−1^ FW h^−1^)	IP	–	3.24 ± 0.20a A	–	2.02 ± 0.06c B	2.64 ± 0.19b A	–	–	–	0.002
	IR	–	3.52 ± 0.23a A	–	2.88 ± 0.04a A	3.66 ± 0.97a A	–	–	–	ns
	DR	–	2.55 ± 0.22ab B	–	3.39 ± 0.45a A	1.49 ± 0.23b A	–	–	–	0.027
*p*			0.0047		0.043	ns				
Malate (nmol g^−1^ FW h^−1^)	IP	–	2.09 ± 0.09a A	–	1.42 ± 0.10b A	2.00 ± 0.15a A	–	–	–	0.003
	IR	–	1.58 ± 0.06a A	–	1.21 ± 0.06a A	0.90 ± 0.21a A	–	–	–	ns
	DR	–	1.38 ± 0.34a A	–	1.25 ± 0.07a A	0.56 ± 0.08a B	–	–	–	ns
*p*			ns		ns	<0.001				
Lactate (nmol g^−1^ FW h^−1^)	IP	–	2.54 ± 0.17a A	–	2.85 ± 0.09a A	2.54 ± 0.23a A	–	–	–	ns
	IR	–	2.51 ± 0.14a A	–	1.62 ± 0.19b B	1.45 ± 0.13b B	–	–	–	0.006
	DR	–	2.51 ± 0.24a A	–	1.38 ± 0.03b B	–	–	–	–	0.01
*p*			ns		<0.001	0.014				

Phosphate uptake rate has been calculated by difference between the concentration of PO_4_^3−^ before and after the contact times of roots with the uptake solution. -P roots have been separated in five different root segments: A, apex; J, juvenile cluster root; R, root; M, mature cluster root; S, senescent cluster root; C roots have been separated in three different root segments: AC, apex control; C, cluster control; RC, root control. Phosphate uptake and carboxylate exudation have been determined using three different root handlings: IP, intact plants; IR, intact roots; DR, dissected roots. Data are presented as mean ± SE, n = 5. Lower case letters indicate statistical significance between different root segments (i.e. A vs J) within the same root handling method according to one-way ANOVA comparing means by Holm-Sidak post hoc test with a significance threshold at p < 0.05. Upper case letters indicate statistical significance within the same root segments but different sampling methods (i.e. IP vs IR) according to one-way ANOVA comparing means by Holm-Sidak post hoc test with a significance threshold at p < 0.05; ns, non-significant.

#### Phosphate Uptake After 4 h

**Table 2** shows the P_i_ uptake of -P and C white lupin roots 4 h after the onset of the light. It is interesting to note significant differences just in roots belonging to white lupins grown in a full nutrient solution (C), with all three root handlings (IP, IR, DR). In AC rootlets, the significant highest P_i_ uptake rate was detected when roots were dissected into single segments prior to the uptake experiment (DR), while it resulted lowest when the roots were separated from the plants without cutting them into smaller pieces (IR, **Table 2**). A similar trend could be observed in cluster rootlets C plants. Again, the single dissected root segments (DR) revealed the highest P_i_ uptake rate compared to roots sampled with the other two root handling set-ups (DR *vs* IR + 273%; DR *vs* IP + 826%). The P_i_ uptake rate in RC rootlets showed the same tendency: P_i_ uptake rate resulted highest in dissected root segments (DR *vs* IR +337%; DR *vs* IP + 283%). When looking at the differences among the root tissues types, only the intact roots (IR) handling method displayed significant differences. The P_i_ uptake rate was the highest in R rootlets, together with A and RC rootlets. On average they showed a 287% higher P_i_ uptake rate compared to all the other root tissues (J, M, S, AC, C).

**Table 2:** Phosphate uptake and citrate, malate and lactate exudation of white lupin roots grown in either -P or C conditions 4 h after the onset of light.

		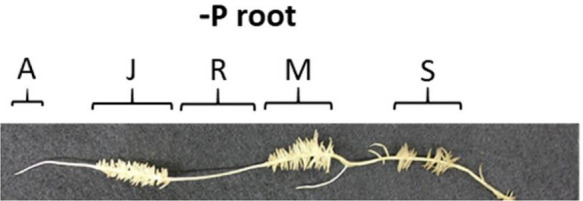 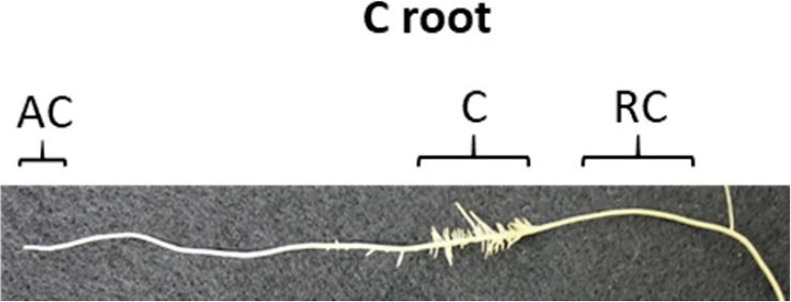								
4h	Sampling	Root segment								
		A	J	R	M	S	AC	C	RC	*p*
P_i_ uptake (µmol g^−1^ FW h^−1^)	IP	0.89 ± 0.12a A	2.79 ± 1.09a A	0.58 ± 0.32a A	1.57 ± 0.14a A	0.94 ± 0.16a A	5.49 ± 0.68a B	0.60 ± 0.19a B	2.83 ± 1.5a B	ns
	IR	3.54 ± 0.61a A	1.75 ± 0.41bc A	3.75 ± 0.75a A	1.09 ± 0.30bc A	0.51 ± 0.14c A	0.76 ± 0.14c C	1.49 ± 0.32bc B	2.48 ± 0.57ab B	<0.001
	DR	6.79 ± 2.96a A	4.58 ± 1.21a A	3.51 ± 0.98a A	1.61 ± 0.42a A	1.11 ± 0.36a A	21.14 ± 2.11a A	5.56 ± 1.45a A	10.85 ± 2.24a A	ns
*p*		ns	ns	ns	ns	ns	< 0.001	0.017	0.017	
Citrate (nmol g^−1^ FW h^v−1^)	IP	–	6.90 ± 0.84a A	–	6.67 ± 0.36a A	3.49 ± 0.16b A	–	–	–	0.002
	IR	–	5.60 ± 0.59a A	–	4.92 ± 0.42a A	3.32 ± 0.58a A	–	–	–	ns
	DR	–	5.91 ± 1.13a A	–	6.58 ± 0.66a A	4.38 ± 0.73a A	–	–	–	ns
*p*			ns		ns	ns				
Malate (nmol g^−1^ FW h^−1^)	IP	–	1.67 ± 0.31b A	–	2.50 ± 0.37b A	5.70 ± 0.32a A	–	–	–	0.003
	IR	–	2.84 ± 0.70a A	–	1.67 ± 0.34a A	2.99 ± 0.19a B	–	–	–	ns
	DR	–	4.08 ± 0.86a A	–	2.23 ± 0.46a A	1.69 ± 0.07a C	–	–	–	ns
p			ns		ns	<0.001				
Lactate (nmol g^−1^ FW h^−1^)	IP	–	3.93 ± 1.04a A	–	6.02 ± 1.34a A	3.70 ± 0.88a A	–	–	–	ns
	IR	–	4.54 ± 0.88a A	–	3.72 ± 1.14ab A	2.17 ± 0.54b A	–	–	–	0.006
	DR	–	4.72 ± 1.16a A	–	2.94 ± 0.28b A	–	–	–	–	0.01
*p*			ns		ns	ns				

Phosphate uptake rate has been calculated by difference between the concentration of PO_4_^3-^ before and after the contact times of roots with the uptake solution. -P roots have been separated in five different root segments: A, apex; J, juvenile cluster root; R, root; M, mature cluster root; S, senescent cluster root. C roots have been separated in three different root segments: AC, apex control; C, cluster control; RC, root control. Phosphate uptake and carboxylate exudation have been determined using three different root handlings: IP, intact plants; IR, intact roots; DR, dissected roots. Data are presented as mean ± SE, n = 5. Lower case letters indicate statistical significance between different root segment (i.e. A vs J) within the same root handling method according to one-way ANOVA comparing means by Holm-Sidak post hoc test with a significance threshold at p < 0.05. Upper case letters indicate statistical significance within the same root segment but different sampling methods (i.e. IP vs IR) according to one-way ANOVA comparing means by Holm-Sidak post hoc test with a significance threshold at p < 0.05; ns, non-significant.

#### Phosphate Uptake After 8 h

**Table 3** reports the P_i_ uptake of white lupin rootlets 8 h after the onset of light. The experimental set-up had a greater effect on the P_i_ uptake at this time point. Besides the J cluster roots, all root tissue types were significantly affected by the root handling in both full and P-deficient nutrient status. In -P conditions, dissected root apices (DR, roots type: A) displayed a 753% higher uptake rate when compared to the apices of the whole root separated from the shoots (IR). The same trend could be observed in mature cluster roots (M roots: +295% DR *vs* IR; +52.4% DR *vs* IP). Furthermore, also senescent rootlets (S) presented the significantly highest P uptake rate in the dissected root segments (IR) having a 362% and 517% higher rate compared to entire roots separated from the shoots (IR) and the roots from intact plants (IP), respectively. The R root tissue type of -P lupin plants displayed the opposite trend showing the highest uptake rate when root segments were not excised (IP and IR experimental set-ups: +304% IP *vs* DR; +74% IR *vs* DR). The apices of control white lupin plants (AC) exhibited a similar P_i_ uptake pattern: apices of intact plants showed the highest P_i_ uptake rate (+1317% IP *vs* IR; +168% IP *vs* DR). On the other hand, P_i_ uptake resulted highest in C rootlets of the excised root segments (+531% DR *vs* IR). Again, RC rootlets exhibited a 297% higher P uptake rate when the roots were dissected in segments rather than kept as entire roots (DR *vs* IR). Concerning the effect on the P_i_ uptake of the root tissue types, significant differences could be observed only in the sampling method in which the plants were kept intact (IP). The significantly highest uptake rate was discovered in the R tissue type (-P plants), the lowest in the S tissue type (-P plants, R *vs* S +3626%). All the other root zones (A, J, M, AC, C, RC) were between those two without any statistical significance.

**Table 3:** Phosphate uptake and citrate, malate and lactate exudation of white lupin roots grown in either -P or C conditions 8 h after the onset of light.

		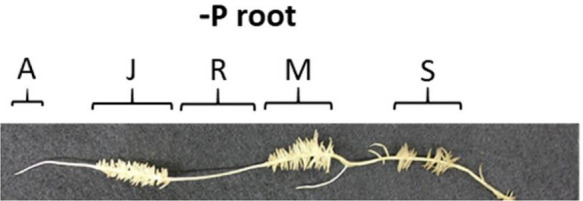 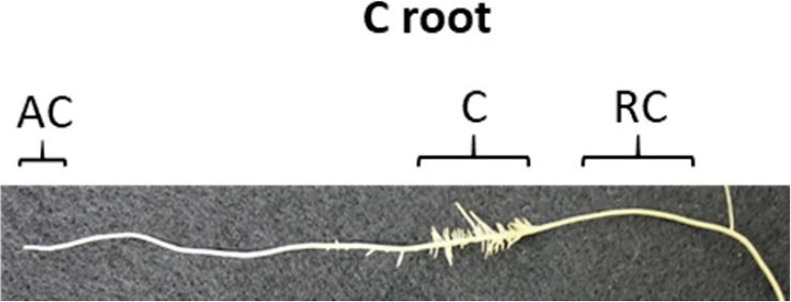								
8h	Sampling	Root segment								
		A	J	R	M	S	AC	C	RC	*p*
P_i_ uptake (µmol g^−1^ FW)	IP	10.94 ± 6 ab A	0.37 ± 0.22ab A	12.33 ± 3.68a A	1.66 ± 0.40ab B	0.34 ± 0.07b B	8.93 ± 0.65ab A	6.29 ± 2.9ab AB	3.93 ± 2.1ab AB	0.013
	IR	1.48 ± 0.37a B	0.54 ± 0.17a A	3.05 ± 0.59a B	0.64 ± 0.15a B	0.45 ± 0.04a B	0.63 ± 0.47a C	1.22 ± 0.56a B	2.36 ± 0.93a B	ns
	DR	12.63 ± 0.79a A	1.39 ± 0.36a A	1.73 ± 0.79a C	2.53 ± 0.44a A	2.08 ± 0.73a A	3.32 ± 0.07a B	7.71 ± 3.70a A	9.37 ± 2.28a A	ns
*p*		0.013	ns	< 0.001	0.009	0.009	< 0.001	0.023	0.023	
Citrate (nmol g^−1^ FW h^v1^)	IP	–	3.88 ± 0.47a B	–	2.24 ± 0.45b B	1.72 ± 0.24b B	–	–	–	0.002
	IR	–	3.34 ± 0.45a B	–	2.81 ± 0.24a B	3.85 ± 0.58a AB	–	–	–	ns
	DR	–	6.39 ± 0.32a A	–	4.39 ± 0.12b A	4.74 ± 0.49ab A	–	–	–	0.027
*p*			0.013		0.002	0.024				
Malate (nmol g^−1^ FW h^−1^)	IP	–	3.27 ± 0.75a A	–	1.51 ± 0.25b A	1.59 ± 0.47b A	–	–	–	0.003
	IR	–	2.04 ± 0.38a A	–	1.09 ± 0.28a A	1.37 ± 0.13a A	–	–	–	ns
	DR	–	3.58 ± 1.16a A	–	1.53 ± 0.17a A	–	–	–	–	ns
*p*			ns		ns	ns				
Lactate (nmol g^−1^ FW h^−1^)	IP	–	5.14 ± 0.44a A	–	2.47 ± 0.34b A	2.47 ± 0.96b A	–	–	–	ns
	IR	–	2.47 ± 0.52a A	–	2.32 ± 0.47a A	3.07 ± 0.95a A	–	–	–	ns
	DR	–	5.06 ± 0.57a A	–	2.77 ± 0.34b A	–	–	–	–	0.01
*p*			ns		ns	ns				

Phosphate uptake rate has been calculated by difference between the concentration of PO_4_^3-^ before and after the contact times of roots with the uptake solution. -P roots have been separated in 5 different root segments: A = apex, J = juvenile cluster root, R = root, M= mature cluster root, S = senescent cluster root. C roots have been separated in 3 different root segments: AC = apex control, C = cluster control, RC = root control. Phosphate uptake and carboxylate exudation have been determined using three different root handlings: IP = intact plants, IR = intact roots, DR = dissected roots. Data are presented as mean ± SE, n = 5. Lower case letters indicate statistical significance between different root segments (i.e. A vs J) within the same root handling method according to one-way ANOVA comparing means by Holm-Sidak post hoc test with a significance threshold at p < 0.05. Upper case letters indicate statistical significance within the same root segment but different sampling methods (i.e. IP vs IR) according to one-way ANOVA comparing means by Holm-Sidak post hoc test with a significance threshold at p < 0.05; ns = non-significant.

### Release of Carboxylates

#### Release of Carboxylates After 1 h

**Table 1** reports the carboxylate exudation of both C and -P lupin plants detected in the collection solution of the different root zones after 1 h from the onset of light. Carboxylate analysis revealed the presence of citrate, malate and lactate in the collection solution of white lupin roots. Citrate, malate and lactate have been detected only in juvenile (J), mature (M) and senescent (S) roots of -P plants.

Citrate exudation of J and M roots was affected by the type of root handling (**Table 1**). Juvenile roots exhibited a significantly higher exudation rate when roots were not dissected in segments (+ 32.5% IP and IR *vs* DR). On the contrary, citrate release from M roots was highest when roots were either dissected in segments or separated from the shoots (+ 67.3% DR *vs* IP; + 42.5% IR *vs* IP). Citrate exudation from S roots was not significantly influenced by the root handling method. When comparing the citrate exudation of the tissue types, significant variations were found in the IP and DR handling method. In the first one J cluster roots showed the highest release of citrate, followed by S roots and finally M cluster roots. In the handling method in which the roots were dissected completely into pieces prior to the sampling (DR), the highest citrate release was observed in M rootlets compared to S rootlets (M *vs* S +227%).

Interestingly, S roots revealed a root handling -dependent exudation pattern for malate (**Table 1**). Indeed, malate exudation resulted highest when roots were not excised in segments: S roots exhibited a 4-fold and 2-fold higher malate exudation rate from intact plants (IP) and roots separated from their shoots (IR), respectively, compared to the dissected S roots (DR). Moreover, concerning the differences of the root tissue types only S and J rootlets displayed a significant higher malate exudation compared to M roots, yet only in the IP root handling method. On average S and J roots showed a 44% higher malate exudation rate compared to M rootlets.

Lactate revealed a completely different pattern compared to citrate and malate (**Table 1**). In particular, M roots released higher lactate rates when roots were not dissected nor separated from their shoots (+75.9% IP *vs* IR, +106% IP *vs* DR). Additionally, S roots showed a significant 75.2% higher lactate exudation rate from roots of the intact plant (IP) when compared to the roots which were separated from their shoots (IR). It is interesting to note that lactate was not detected in excised S roots (**Table 1**). Regarding the changes of lactate exudation among different root tissue types significant differences could be found in the two handling methods in which the roots were cut (IR, DR). In both cases, the significantly highest lactate release was observed in J roots compared to M and S roots (IR: +53% J *vs* M/S; +81% DR: J *vs* M).

#### Release of Carboxylates After 4 h

**Table 2** reports the carboxylate exudation detected in the collection solution of the different root segments after 4 h from onset of light of the climate chamber of both white lupin plants raised in C and -P nutrient conditions. Analysis of the carboxylates revealed again the presence of citrate, malate, and lactate in the collection solution of white lupin roots. However, those compounds were found only in the following root segments of -P plants: juvenile (J), mature (M), senescent (S).

Citrate exudation was not affected by the type of root handling (**Table 2**). On the other hand, citrate exudation was influenced by root tissue type in the intact plant (IP) set-up. Juvenile and M cluster roots both exuded significantly more citrate than senescent (S) rootlets (on average +94%).

Malate exudation revealed a different pattern compared to citrate (**Table 2**). Especially, S rootlets showed a significant higher malate exudation when the plant was kept intact (IP), followed by the IR handling method and lastly the completely dissected roots (DR). The IP set-up in S rootlets showed a 90.6% higher malate release when compared to the IR set-up, while 237% higher with respect to the DR set-up. Malate exudation was affected by the root tissue type only in the IP handling set-up. The S roots showed the highest malate release when compared to both J and M rootlets (+ 241% M and S *vs* J; + 128% S *vs* M).

Lactate exudation of white lupin roots 4 h after the onset of light was not altered significantly by the root handling methods (**Table 2**). On the other hand, the different root tissue types showed a significant effect on the lactate detected in the collection solution of the IR and DR roots. In the IR handling method, the J cluster roots displayed the significant highest release of lactate when compared to the senescent rootlets (+109% J *vs* S). The same could be observed in the completely dissected roots handling method (DR). Juvenile cluster roots showed a 60% higher lactate release rate compared to M rootlets. It is interesting to note that no lactate was detected in the exudate collection solution of S rootlets in the DR handling method.

#### Release of Carboxylates After 8 h

**Table 3** reports the carboxylate exudation of both C and -P lupin plants detected in the collection solution of the different root segments after 8 h from onset of light. The carboxylate analysis of the exudate solutions revealed the presence of citrate, malate, and lactate in juvenile (J), mature (M) and senescent (S) rootlets. Carboxylates were not detectable in the collection solutions of control (C) roots.

Citrate exudation of J roots was significantly altered by the root handling (**Table 3**). The completely dissecting of roots (DR) showed a significantly higher (91.3%) citrate exudation rate compared to the set-up in which roots were cut only once (IR setup) and a 64.6% higher rate compared to intact plants (IP set-up). The same trend was observed in M rootlets (+56.2% DR *vs* IR; +95.9% DR *vs* IP), as well as in S roots (+175% DR *vs* IP). Citrate exudation was significantly affected by the different root segments within the same handling methods. In the IP- set-up, J rootlets exuded the significantly highest citrate rate when compared to both M and S cluster roots (+75.5% J *vs* M; +125%J *vs* S). Furthermore, a similar trend could be observed in the handling method in which the roots were dissected completely into small segments (DR). Also, with this root handling method, the J rootlets displayed the significantly highest citrate exudation rate when compared to M cluster rootlets (+ 45% J *vs* M).

Malate exudation showed a completely different pattern than citrate exudation (**Table 3**). The root handling methods (IP, IR, DR) revealed no significant effect on the malate exudation. However, in the set-up in which the plant was kept intact (IP) an effect of the different root tissue types could be observed. Very similar to citrate, malate release resulted highest in juvenile (J) cluster root compared to both mature (M) and senescent (S) rootlets (+116% J *vs* M, +105% J *vs* S). No significant effect of the root tissue type was found in the other two handling methods (IR, DR). Interestingly, no malate was detected in the exudate collection solution of S rootlets in the completely dissected roots (DR).

Lactate exudation showed a similar trend as malate (**Table 3**). The root handling methods (IP, IR, DR) revealed no significant effect on the lactate exudation. On the other hand, an effect of the different root tissue types could be observed in the handling method in which lupin roots were dissected completely into pieces (DR). The J rootlets displayed an 82% higher exudation rate than M roots. Similarly, to malate, no lactate was detected in the exudate collection solution of S rootlets in the completely dissected roots handling type (DR).

## Discussion

### Plant Morphology and General P Deficiency Symptoms

This study used hydroponically grown white lupin plants together with a custom-made device (**Figures 1** and **2**) to examine how different root handling methods influenced both P_i_ uptake and carboxylate exudation of single root segments when plants were subjected to either P sufficient or lacking conditions. The study used -P plants to enhance the root exudation and stimulate the development of cluster roots in white lupin. The concentration of 100 µmol L^−1^ P_i_ was used to measure the potential capacity of P_i_ uptake considering both low and high affinity P_i_ transport systems (Furihata et al., 1992; Wang et al., 2017). The -P white lupins used in this research showed all the typical symptoms of P deficiency (**Figure 3**). Generally, well known symptoms of P deficiency in plants include an increased root-to-shoot ratio (López-Arredondo et al., 2014; Pandey et al., 2015), as well as alterations of the root morphology (Neumann and Martinoia, 2002; López-Arredondo et al., 2014). In fact, the -P plants in this study presented a 42% higher shoot to root ratio than C plants (**Figure 3A**). As expected, white lupin plants produced huge amounts of cluster roots to cope with P deficiency (Uhde-Stone, 2017) (**Figure 3E**). Cluster root formation is very complex and probably modulated by signaling hormones (*e.g.* auxin, nitric oxide), sucrose and miRNAs (*e.g.* miR399) (Liu and Vance, 2010; Wang et al., 2015; Uhde-Stone, 2017; Zhou et al., 2019). Additionally, cluster roots can be also formed in plants with sufficient P nutrition, although those cluster roots are not fully functional (Meng et al., 2013; Wang et al., 2015). Indeed, also this study was able to identify cluster root like structures in control plants (**Figure 3D**). Cluster roots do not only exude huge amounts of carboxylates but also intensely secret protons to solubilize the scarcely soluble P_i_ (Wang et al., 2006; Uhde-Stone, 2017). Qualitative visualization of pH changes due to proton exudation was also performed in this study and confirms these findings. Indeed, **Figure 3C** shows that -P white lupin roots acidify (yellow coloration) the rhizosphere to a much greater extent compared to C white lupin roots (**Figure 3B**).

### Phosphate Uptake and Carboxylate Exudation

A very important aspect in the context of this study is the presentation of the data, especially in terms of units (Oburger and Jones, 2018). The results were normalized (divided) by the roots´ fresh weight. Normalization on root surface area or root length would completely bias the results since cluster roots of -P roots (M, J, S rootlets) generally have a much higher fresh weight. Moreover, reproducible measurements of the roots surface area is complex and the literature generally recommends to normalize by the root weight (Oburger and Jones, 2018). On the other hand, since the weight of root tissue types such as A, R and AC and RC is much smaller than the cluster roots the P_i_ uptake/carboxylate exudation is more pronounced when indicating the data per root fresh weight.

Since the aim of the study was to assess the difference between the root handling methods, one-way ANOVAs were chosen as statistical tests over pairwise comparisons. Even though pairwise comparisons could lead to more significant results, ANOVAS highlight the most relevant differences more suitable to the aim of the study.

#### Phosphate Uptake

Phosphate uptake is a crucial process for plants. Compared to other major nutrients (such as nitrogen, potassium etc.), P_i_ is far less mobile and thus more difficult for plants to acquire (Hinsinger, 2001). Especially when growing in P-deficient soils, plants must adapt in order to acquire all the available P_i_. The measurement of plants´ rhizosphere processes such as root P_i_ uptake is methodologically very challenging (Oburger and Schmidt, 2016; Oburger and Jones, 2018), and even more so when targeting single root segments. Most studies which targeted root segments worked with excised roots (Vorster and Jooste, 1986; Massonneau et al., 2001) or with radioactive labeled ^32^P (Kreuzwieser et al., 1997; Flatian et al., 2018; Cruz-Paredes and Gavito, 2020). Furthermore, there is a lot of research which used excised roots (Benlloch et al., 1983; Bloom and Caldwell, 1988; Tu et al., 1990; Rubio et al., 2004; Harley and McCready, 2006; Lucash et al., 2007; Abbas and Meharg, 2008; Horbowicz et al., 2011). Root handling methods such as dissecting/cutting/excising/may disturb roots and thus artificially increase or decrease the net uptake (Bloom and Caldwell, 1988; Oburger and Schmidt, 2016). The present study revealed that the root handling influences the P_i_ uptake rate of white lupin roots. However, the effect was limited to 4 and 8 h after the onset of light (**Tables 2** and **3**). No effect occurred 1 h after the onset of light (**Table 1**). White lupin plants are known to have a time-dependent citrate exudation pattern (Dessureault-Rompré et al., 2007; Tomasi et al., 2009; Mimmo et al., 2011), releasing the main citrate burst around 4 to 5 h after the onset of light (Tomasi et al., 2009). Since the roots are probably more active at that time, it is most likely that the P_i_ uptake was not influenced in the first sampling time point (1 h). Four hours after the onset of light, effects of the root handling methods on the P_i_ uptake could be detected (**Table 2**), yet only in roots of plants grown in a full NS (AC, C, RC tissue). Roots completely dissected into pieces (DR) displayed in all three root tissue types the highest uptake rate. This indicates that the complete dissection increases the P_i_ uptake greatly and can lead to biased data. A study conducted on cut maize roots with ^32^P came to the same result, showing that the excision can enhance the P_i_ influx (Gronewald and Hanson, 1980). In contrast to that, root dissection has been shown to decrease NO_3_^-^ (Aslam et al., 1996) and NH_4_^+^ uptake (Bloom and Caldwell, 1988). The sampling at 8 h after the onset of the light led to the most significant alterations of the root P_i_ uptake (**Table 3**) induced by the sampling method. In some cases, a similar trend as at 4 h could be observed with the DR sampling method showing the highest uptake in most tissues (A, M, S, C, RC). Though, also the IP method showed a high P_i_ uptake rate (**Table 3**). The dissection of the roots, both complete or just partial (IR, DR), has a huge impact and thus it could be expected to have a great influence on physiological parameters (*e.g.* P_i_ uptake). Nevertheless, the influence of the root handling on P_i_ uptake shows that the results should be carefully interpreted.

The A rootlet tissue type displayed in most cases the highest uptake when normalized per fresh weight. Therefore, it is supposed that the root apex/tip could act as a sensor in order to scout for nutrients. If no nutrients are sensed the tip triggers a signal cascade in order to cope with the nutrient deficiency, *e.g.* developing clusters and increasing exudation to enhance the availability of P_i_. In fact, it has been already postulated by some authors in the case of *Arabidopsis thaliana* (Abel, 2017) and confirmed in maize (*Zea mays* L.) roots (Trevisan et al., 2015). Moreover, it might be hypothesized that root tips could sense concentration changes of exuded metabolites and translate this into signals to modify root growth (Canarini et al., 2019), enforcing the theory that the tip/apex of the root acts as a kind of sensor for the plant.

#### Carboxylate Exudation

Exudation of carboxylates represents one of the most common responses to nutrient deficiencies in plants (Vives-Peris et al., 2019). Exudation of carboxylates (citrate, malate and lactate) was affected significantly by the root handling method in the samplings 1 and 8 h after the onset of light (**Tables 1** and **3**). At 1 h after the onset of light the roots of intact plants (IP) had the tendency to exude the highest concentration of carboxylates (**Table 1**). In this case, the data suggest that the root dissection decreases the exudation rather than increasing it. Indeed, we rather hypothesized that the physical damage to the roots leads to a higher exudation. To the best of our knowledge, no scientific literature is available about the effect of root cutting/dissecting on root exudation. The only evidence about the effect of physical damage on root exudation is rather dated. These authors found that amino acids release from damaged wheat and pea roots leads to 73% to 120% of that released by intact roots (Ayers and Thornton, 1968). The sampling performed at 4 h after the onset of light had just minor effects on the exudation (**Table 2**). The lack of major significant alterations of the exudation rate at the 4-h sampling time point (at noon) could be explained again (as in section 4.2.1) by the time-dependent citrate exudation pattern (Mimmo et al., 2011). The higher root activity at noon could counteract the disturbing effects of the root dissection. At 8 h after the onset of light only citrate exudation was affected by the root handling method (**Table 3**). However, the opposite trend was observed compared to the exudation at 4 h after the onset of light. The completely dissected roots showed the highest citrate exudation rate. For the first time, this study quantifies the effects of root handling/cutting/dissecting on the exudation of carboxylates. Although no clear and distinctive trend could be observed, we revealed that the handling and the sampling time point can greatly influence the outcomes. Undeniably, these two factors should not be neglected when interpreting rhizosphere dynamics.

## Conclusion

The present study examined for the first time the effect of three different root handling methods on the P_i_ uptake and carboxylate exudation of different cluster root zones of white lupin plants grown hydroponically in a full and P-deficient nutrient solution. The results imply that the handling method influences greatly both P_i_ uptake and carboxylate exudation and thus has to be considered when studying roots in a destructive way. Moreover, the study revealed, that in plant species like white lupins, which shows a diurnal pattern of root activity, the effect of the root handling depends on sampling time point. Additionally, the study enforced the growing evidence that the root tip acts as a sensor for P_i_.

## Data Availability Statement

The raw data supporting the conclusions of this article will be made available by the authors, without undue reservation.

## Author Contributions

TM, StC, YP, and RT designed the study. RT, FV, and SiC performed the experiments. RT, TM, and StC wrote the paper. All authors contributed to the article and approved the submitted version.

## Funding

This work was supported by grants from the Free University of Bolzano (NUMICS TN200E). The doctoral fellowship of RT was funded by Stiftung Südtiroler Sparkasse.

## Conflict of Interest

The authors declare that the research was conducted in the absence of any commercial or financial relationships that could be construed as a potential conflict of interest.

The handling Editor declared a past co-authorship with three of the authors, TM, SiC, and StC.
