# Corrigendum: Root Handling Affects Carboxylates Exudation and Phosphate Uptake of White Lupin Roots

**DOI:** 10.3389/fpls.2021.681263

**Published:** 2021-04-22

**Authors:** Raphael Tiziani, Tanja Mimmo, Fabio Valentinuzzi, Youry Pii, Silvia Celletti, Stefano Cesco

**Affiliations:** Faculty of Science and Technology, Free University of Bolzano, Bolzano, Italy

**Keywords:** carboxylates, cluster roots, phosphate uptake, root exudates, white lupin

In the original article, the authors neglected to acknowledge the funding provided by “Stiftung Südtiroler Sparkasse” (to Raphael Tiziani). The **Funding** section in the published article has been updated as presented below.

This work was supported by grants from the Free University of Bolzano (NUMICS TN200E). “The doctoral fellowship of RT was funded by Stiftung Südtiroler Sparkasse.”

The authors apologize for this error and state that this does not change the scientific conclusions of the article in any way. The original article has been updated.

